# Secondary Bacterial Infection and Clinical Characteristics in Patients With COVID-19 Admitted to Two Intensive Care Units of an Academic Hospital in Iran During the First Wave of the Pandemic

**DOI:** 10.3389/fcimb.2022.784130

**Published:** 2022-02-23

**Authors:** Samaneh Pourajam, Elham Kalantari, Hamid Talebzadeh, Hamid Mellali, Ramin Sami, Forogh Soltaninejad, Babak Amra, Mahdi Sajadi, Malihe Alenaseri, Forough Kalantari, Hamid Solgi

**Affiliations:** ^1^ Department of Internal Medicine, School of Medicine, Isfahan University of Medical Sciences, Isfahan, Iran; ^2^ Department of Pulmonology, Isfahan University of Medical Science, Isfahan, Iran; ^3^ Department of Surgery, School of Medicine, Isfahan University of Medical Sciences, Isfahan, Iran; ^4^ The Respiratory Research Center, Pulmonary Division, Department of Internal Medicine, School of Medicine, Isfahan University of Medical Sciences, Isfahan, Iran; ^5^ Bamdad Respiratory Research Center, Isfahan University of Medical Sciences, Isfahan, Iran; ^6^ Division of Clinical Microbiology, Department of Laboratory Medicine, Amin Hospital, Isfahan University of Medical Sciences, Isfahan, Iran; ^7^ Infection Control Unit of Amin Hospital, Isfahan University of Medical Sciences, Isfahan, Iran; ^8^ Department of Nuclear Medicine, Rasoul Akram Hospital, Iran University of Medical Sciences, Tehran, Iran; ^9^ Isfahan Endocrine and Metabolism Research Center, Isfahan University of Medical Sciences, Isfahan, Iran

**Keywords:** bacterial co-infection, antibiotic use, COVID-19, carbapenem resistant bacteria, intensive care unit

## Abstract

Data on the prevalence of bacterial co-infections and secondary infection among adults with COVID-19 admitted to the intensive care unit (ICU) are rare. We aimed to determine the frequency of secondary bacterial infection, antibiotic use, and clinical characteristics in patients admitted to the ICU with severe SARS-CoV-2 pneumonia. This was a retrospective cohort study of adults with severe COVID-19 admitted to two ICUs from March 6 to September 7, 2020 in an academic medical center in Isfahan, Iran. To detect COVID-19, reverse transcription real-time polymerase chain reaction was performed and also typical pattern of CT scan was used for the diagnosis of COVID-19. Data collection included the age, gender, main symptoms, history of underlying disease, demographics, hospital stay, outcomes, and antibiotic regimen of the patient. Antimicrobial susceptibility testing was carried out according to the CLSI guidelines. During the study period, 553 patients were referred to the both ICUs for COVID-19 with severe pneumonia. Secondary bacterial infection was detected in 65 (11.9%) patients. The median age was 69.4 (range 21–95) years; 42 (63.6%) were men. Notably, 100% (n = 65) of the patients with superinfection were prescribed empirical antibiotics before first positive culture, predominantly meropenem (86.2%) with a median duration of 12 (range 2–32) days and levofloxacin (73.8%) with a median duration of nine (range 2–24) days. Most prevalent causative agents for secondary bacterial infection were *Klebsiella pneumoniae* (n = 44) and *Acinetobacter baumannii* (n = 33). Most patients with secondary bacterial infection showed extensive drug-resistance. The mortality among patients who acquired superinfections was 83% against an overall mortality of 38.1% in total admitted COVID-19 patients. We found a high prevalence of carbapenem-resistant Gram-negative bacilli in COVID-19 patients admitted to our ICUs, with a high proportion of *K. pneumoniae* followed by *A. baumannii*. These findings emphasize the importance of implementation of strict infection control measures and highlight the role of antimicrobial stewardship during a pandemic.

## Introduction

The severe acute respiratory syndrome coronavirus 2 (SARS-CoV-2), which causes coronavirus disease 2019 (COVID-19), was first identified in December 2019 in Wuhan, China, and is currently circulating worldwide. COVID-19 disease is the greatest pandemic of our generation, with 227 million people infected and 4,664,000 deaths worldwide (WHO). The World Health Organization (WHO) guidelines for the clinical management of COVID-19 disease advise clinicians to collect specimens from the upper respiratory tract and blood samples for bacterial cultures, and to start empirical antibiotic therapy only in severe cases (WHO, Interim guidance March 13, 2020). It is becoming apparent that secondary bacterial infections occur in many COVID-19 patients and lead to increase disease severity and mortality, especially in those requiring invasive mechanical ventilation. Bacterial co-infection and secondary infection rates are generally low in COVID-19 patients, with higher rates in critically ill intensive care unit (ICU)-patients. Many studies of COVID-19 patients admitted to the ICU note the empiric use of antibiotics in a majority of patients, which can lead to increases in the prevalence of multidrug-resistant (MDR) bacteria (Contou et al., 2020; Sharifipour et al., 2020; Baskaran et al., 2021). COVID-19 pneumonia is associated with high rates of admission to the ICU and in-hospital mortality (Contou et al., 2020). Among COVID-19 patients, the bacteria, namely, *Klebsiella pneumoniae*, *Acinetobacter* spp., and *Pseudomonas* spp., *Escherichia coli*, and *Staphylococcus* spp., are the most frequently detected causative pathogens (Sharifipour et al., 2020). Understanding the proportion of COVID-19 patients with acute respiratory bacterial co-infection is crucial for treating patients with COVID-19 and to help ensure responsible use of antibiotics for decreased rates of antibiotic-resistant bacteria (Russell et al., 2021). We aimed to assess the rate of secondary bacterial infections, their antibiotic resistance, antibiotic therapies and clinical characteristics applied among patients admitted to our hospital for severe SARS-CoV-2 pneumonia in Isfahan, Iran.

## Materials and Methods

### Data Collection

We conducted a single-center retrospective study including all adult (≥18 years old) patients admitted to two COVID-19 ICUs from March 6 to September 7, 2020 in an academic hospital with approximately 220 beds, located in in Isfahan, Iran, for acute respiratory failure related to SARS-CoV-2 pneumonia. In this study, the criteria for admission to the ICU wards was a positive RT-PCR test or presence of ground glass opacity in the CT-scan. The collected clinical data include: age, gender, main symptoms, history of underlying disease, patient demographics, hospital stay, and outcomes and also the antibiotic and steroid regimen of the patient.

### Reverse Transcription Real-Time Polymerase Chain Reaction (RT-PCR) for the Detection of COVID-19

This step was performed once for each patient. Briefly, nasopharyngeal and tracheal aspirate samples were taken from the patients and then placed in a separate collection tube containing 2–3 ml of viral transport medium and immediately sent to the COVID-19 reference laboratory of the university. RNA extraction was performed using the Viral Nucleic Acid Extraction kit according to the manufacturer’s protocol (RBC Bioscience, Taipei, Taiwan). RT-PCR was performed using LightMix^®^ Modular SARS and Wuhan CoV E-gene kit and using one-step RT-PCR polymerase Mix (Tib-Mol-biol, Berlin, Germany) (Corman VM et al., 2020).

### Collection of Clinical Specimens for Bacterial Detection

In the present study, over six months, 365 clinical samples such as blood, catheters, cerebrospinal fluid (CSF), sputum, stool, tracheal aspirate, wound and urine were collected from inpatients in two different ICU wards of an educational hospital affiliated to the Isfahan University of Medical Sciences, Isfahan, Iran. The clinical samples were cultured on Blood agar, Chocolate Agar and MacConkey agar (Merck) and then incubated at 37°C for 24–48 h under standard conditions. Species identification was carried out using standard biochemical tests and API 20E (bioMérieux, Marcy-l’Etoile, France). In addition, *Acinetobacter baumannii* species were confirmed for *bla*_OXA-51_ gene by PCR (Turton et al., 2006).

### Antimicrobial Susceptibility Testing and MIC Determination

Antimicrobial susceptibility testing was performed using the disc diffusion method according to the Clinical and Laboratory Standards Institute [CLSI] guidelines. The antibiotics tested included ceftazidime (CAZ), ceftriaxone (CTX), cefepime (CPM), ampicillin/sulbactam (SAM), piperacillin/tazobactam (PTZ), meropenem (MEM), amikacin (AM), gentamicin (GM), ciprofloxacin (CIP), and levofloxacin (LEV). The minimal inhibitory concentration (MIC) of meropenem was determined by gradient test strips (Liofilchem, Italy) based on the CLSI guideline (CLSI, 2017). MICs of colistin were determined by broth macrodilution method using colistin sulfate (Sigma-Aldrich), and EUCAST breakpoints were used for interpretation (EUCAST, 2017) (EUCAST). *E. coli* ATCC 25922 and *S. aureus* ATCC 29213 were used as quality control strains.

### Definitions

A positive COVID-19 case was defined as a person with a confirmed positive result on a nasopharyngeal and tracheal swab tested using RT-PCR testing for SARS-CoV2 and also the presence of specific COVID-19 CT features. Secondary infections were identified by bacterial infection that developed during ICU stay but after admission of more than 48 h duration, meaning not present at the time of presentation with COVID-19. Secondary bacterial infections in COVID-19 patients are typically referred to as superinfections. Extensively drug resistant (XDR) was defined as non-susceptibility to at least one agent in all but two or fewer antimicrobial categories (i.e., bacterial isolates remain susceptible to only one or two available classes).

### Statistical Analysis

Continuous data are described by median (range) and categorical data by absolute and relative frequencies. The categorical variables were denoted as absolute and relative values (frequencies) and compared by Fisher’s exact Chi-square test using P <0.05 as the level of significance. Statistical analyses were performed using Microsoft Excel 2007 and IBM SPSS Statistics Version 23.0.

## Results

During the study period, a total of 553 adult patients were admitted to our ICU wards (ICU-1 N = 439 and ICU-2 N = 114) for acute respiratory failure due to SARS-CoV-2 pneumonia. Bacterial and fungal positive culture results showed that sixty-five out of 553 patients (11.9%) were positive for bacterial infections and six out of 553 of patients (1%) had only fungal infections. As shown in **Table 1**, the median age of patients was 69.4 years (range of age 21–95 years), and 42 (63.6%) patients were men. At least one underlying comorbidity was present in 53.8%. Prevalent underlying diseases included heart disease (21.5%; n = 14) and diabetes mellitus (15.3%; n = 10) (see **Table 1**). Approximately the median length of hospital stay and the median duration of ICU stay was 22 and 17 days, respectively. The overall mortality of the total population in both ICUs was 38.1% (211/553) (ICU-1 = 34.8% and ICU-2 = 50.8%). Mortality among COVID-19 patients who acquired secondary bacterial infections was 83% (54/65), whereas mortality rate among patients without bacterial superinfections was 32.1% (211–54/488). There was a significantly higher rate of inpatient mortality (83% vs 32.1%; P <.0001) among patients with concomitant bacterial infection compared to those without. Also, fifty-six out of 65 received corticosteroids to the treatment of COVID19. Nineteen and 26 patients received dexamethasone (6 mg/day) with a median duration of six (1–22) days and methylprednisolone (2 mg/kg/day) with a median duration of four (1–8) days, respectively, and 11 patients received both (see **Table 2**).

**Table 1:** Baseline demographics and clinical characteristics of the study patients.

Characteristics	Value (n = 65) %
**Age median (mean ± SD)**	69.4 (21-95)
**Gender n (%)**	
Female	24 (36.9%)
Male	41 (63.1%)
Comorbidities no treatment for COVID-19*n (%)	
Diabetes	10 (15.3%)
Hypertension	3 (4.6%)
Chronic Kidney disease	5 (7.7%)
COPD	5 (7.7%)
Heart disease	14 (21.5%)
Chronic neurological disease	4 (6.1%)
Any medical immunosuppression	3 (4.6%)
**Symptom duration before admission, n (%)**	
Fever	29 (44.6%)
Cough/shortness of breath	58 (89.2%)
Muscular or joint pain	27 (41.5%)
Nausea and vomiting	8 (12.3%)
Anorexia	20 (30.7%)
Headache	5 (7.7%)
**Radiology no treatment for COVID-19**	
Ground-glass opacity (GGO)	53 (81.5%)
Consolidation	8 (12.3%)
Mix	6 (9.2%)
**COVID‐19 treatment**	
Hydroxychloroquine	25 (38.4%)
Oseltamivir	3 (4.6%)
Kaletra	2 (3%)
**Outcomes**	
Mechanical ventilation	40 (61.5%)
ICU length of stay (days)	17 (4-61)
Hospitalization duration	22 (4-64)
In-hospital mortality	54 (83.1%)
Discharged	11 (16.9%)

*As defined by clinicians in admission records.

**Table 2:** Characteristics of patients hospitalized with coronavirus disease 19 (COVID-19).

Patient	Specimens	Species	Ward	Date of hospitalization	Date of intubation	Sampling date	Length of stay in ICU (Days)	Hospitalization duration	RT-PCR	Antimicrobial susceptibility phenotype	Outcomes	Comorbidities	Treatment/duration (days)*	Steroid/duration (days)
1.	Blood	*A. baumannii *	ICU-1	2020/3/15	NI	2020/3/22	9	10	Pos	SAM, COL	Died	COPD	COL/3	MTP/4
2.	Sputum	*K*. *pneumoniae*	ICU-1	2020/3/9	NI	2020/3/28	61	64	Pos	COL	Survived	Diabetes, Heart disease	COL/10	MTP/6
	Bal	*K*. *pneumoniae*			2020/5/3	2020/5/6				COL				
3.	TA	*A. baumannii *	ICU-2	2020/3/24	2020/3/25	2020/3/27	7	15	Neg	SAM, COL	Survived	-	COL/9	DXM/5
4.	TA	*K*. *pneumoniae*	ICU-1	2020/3/11	2020/3/19	2020/3/29	23	22	Pos	COL	Died	–	COL/1	DXM/5
5.	Sputum	*A. baumannii *	ICU-1	2020/3/15	NI	2020/4/2	22	24	Pos	SAM, COL	Died	Diabetes, Heart disease	COL/4	DXM/5, MTP/2
6.	TA	*K*. *pneumoniae*	ICU-1	2020/3/14	2020/3/20	2020/4/2	26	27	Neg	COL	Died	–	COL/5	MTP/8
7.	TA	*K*. *pneumoniae*	ICU-1	2020/3/25	2020/4/3	2020/4/5	38	41	Neg	AM, COL	Died	Heart disease	COL/3	DXM/2, MTP/4
8.	TA	*A. baumannii *	ICU-1	2020/3/27	2020/3/28	2020/4/5	32	45	Pos	SAM, COL	Survived	–	COL/4	DXM/2, MTP/6
9.	Sputum	*K*. *pneumoniae*	ICU-1	2020/3/25	NI	2020/4/5	9	12	Pos	COL	Died	-	-	DXM/5
10.	TA	*A. baumannii *	ICU-2	2020/3/18	2020/3/30	2020/4/11	23	25	Pos	COL	Died	–	COL/1	DXM/6, MTP/2
11.	TA	*A. baumannii *	ICU-1	2020/4/11	2020/4/17	2020/4/20	10	11	Pos	COL	Died	-	-	MTP/3
12.	TA	*A. baumannii *	ICU-1	2020/4/17	2020/4/19	2020/4/22	30	31	Pos	COL	Died	–	COL/4	DXM/22
	Wound	*K*. *pneumoniae*				2020/4/25				COL				
13.	TA	*A. baumannii *	ICU-1	2020/4/17	2020/4/25	2020/4/25	7	8	Neg	COL	Died	-	-	-
14.	TA	*A. baumannii *	ICU-1	2020/3/25	2020/4/3	2020/4/30	35	41	Neg	SAM, COL	Died	Diabetes, Heart disease	COL/3	DXM/2, MTP/3
15.	Sputum	*K*. *pneumoniae*	ICU-1	2020/5/5	NI	2020/5/10	8	9	Neg	COL	Died	-	COL/1	DXM/2
16.	Blood	*E. coli*	ICU-1	2020/4/30	NI	2020/5/12	11	12	Pos	AM, PTZ, CAZ, MEM, COL,	Died	CKD, COPD, Heart disease	CAZ/10, COL/1	–
17.	Urine	*A. baumannii *	ICU-1	2020/5/4	NI	2020/5/16	20	22	Neg	COL	Died	-	COL/1	DXM/3
18.	TA	*A. baumannii *	ICU-1	2020/5/2	2020/5/2	2020/5/17	25	56	Neg	COL	Survived	Hypertension	COL/34	DXM/1
19.	TA	*K*. *pneumoniae*	ICU-1	2020/3/27	2020/3/27	2020/4/17	30	31	Neg	COL	Died	-	-	DXM/1
20.	TA	*K*. *pneumoniae*	ICU-1	2020/5/14	2020/5/15	2020/5/21	10	11	Pos	AM, GM, MEM, COL	Died	Heart disease	MEM/3, COL/1	DXM/5
	Blood	*K*. *pneumoniae*								AM, COL				
21.	Sputum	*A. baumannii *	ICU-1	2020/5/11	NI	2020/5/21	4	10	Pos	AM, COL	Died	Heart disease	-	MTP/3
		*K*. *pneumoniae*								AM, COL				
22.	Blood	*K*. *pneumoniae*	ICU-1	2020/5/4	NI	2020/5/23	21	32	Neg	AM, COL	Died	–	COL/1	DXM/3
23.	TA	*K*. *pneumoniae*	ICU-1	2020/5/21	2020/5/21	2020/5/24	16	17	Neg	AM, GM, COL	Died	CND	COL/7	MTP/1
24.	Sputum	*A. baumannii *	ICU-1	2020/5/10	NI	2020/5/27	30	31	Pos	COL	Died	–	COL/14	DXM/10
		*K*. *pneumoniae*								COL				
25.	TA	*A. baumannii *	ICU-1	2020/5/11	2020/5/25	2020/5/27	6	16	Pos	SAM, COL	Died	COPD, Heart disease	-	MTP/4
26.	Sputum	*A. baumannii *	ICU-1	2020/5/16	NI	2020/5/27	11	20	Pos	SAM, COL	Died	–	COL/5	–
27.	TA	*A. baumannii *	ICU-1	2020/5/15	2020/5/20	2020/5/30	16	17	Neg	COL	Died	Hypertension	COL/2	-
		*K*. *pneumoniae*								COL				
28.	Sputum	*A. baumannii *	ICU-1	2020/5/20	NI	2020/6/3	9	17	Neg	COL	Died	Heart disease	COL/3	MTP/3
29.	TA	*A. baumannii *	ICU-1	2020/5/31	2020/5/31	2020/6/5	6	7	Pos	COL	Died	Hypertension	-	MTP/4
		*K*. *pneumoniae*								COL				
30.	TA	*A. baumannii *	ICU-2	2020/5/2	2020/5/2	2020/5/6	21	53	Neg	COL	Survived	CND	COL/27	DXM/3
		*K*. *pneumoniae*								COL				
31.	Sputum	*A. baumannii *	ICU-1	2020/5/30	NI	2020/6/9	10	11	Pos	SAM, COL	Died	Heart disease	-	MTP/2
32.	TA	*A. baumannii *	ICU-1	2020/6/2	2020/6/9	2020/6/11	9	10	Pos	SAM, COL	Died	–	–	MTP/4
33.	Sputum	*A. baumannii *	ICU-1	2020/6/9	NI	2020/6/12	3	4	Neg	AM, GM	Died	-	-	-
34.	Sputum	*A. baumannii *	ICU-1	2020/5/19	NI	2020/6/13	24	25	Pos	COL	Died	CND	COL/1	DXM/2, MTP/2
		*K*. *pneumoniae*								COL				
35.	TA	*K*. *pneumoniae*	ICU-2	2020/6/8	2020/6/14	2020/6/17	12	14	Pos	COL	Died	-	COL/4	MTP/5
36.	TA	*P. aeruginosa*	ICU-2	2020/6/3	2020/6/15	2020/6/19	20	30	Neg	AM, GM, PTZ, MEM, COL	Died	–	MEM/12	–
37.	Blood	*S. epidermidis*	ICU-1	2020/5/2	NI	2020/6/22	20	50	Neg	VAN, LEN, TEC	Survived	CND	COL/22, LIN/14	DXM/1
38.	Sputum	*K*. *pneumoniae*	ICU-2	2020/6/7	NI	2020/6/24	18	20	Pos	–	Died	–	COL/4	MTP/7
39.	TA	*K*. *pneumoniae*	ICU-1	2020/6/10	2020/6/15	2020/6/25	14	15	Pos	COL	Died	-	-	MTP/5
40.	Sputum	*A. baumannii *	ICU-1	2020/6/8	NI	2020/6/27	27	28	Neg	COL	Died	MI	COL/14	MTP/3
		*K*. *pneumoniae*								COL				
41.	TA	*K*. *pneumoniae*	ICU-1	2020/6/20	2020/6/28	2020/7/1	11	13	Neg	COL	Died	COPD, MI	-	DXM/5, MTP/3
42.	Sputum	*A. baumannii *	ICU-1	2020/6/23	NI	2020/7/1	10	11	Neg	COL	Died	Heart disease	–	–
		*K*. *pneumoniae*								COL				
43.	Sputum	*K*. *pneumoniae*	ICU-1	2020/6/10	NI	2020/6/25	14	15	Pos	COL	Died	Diabetes	-	MTP/5
44.	Sputum	*K*. *pneumoniae*	ICU-1	2020/6/23	NI	2020/7/5	16	26	Pos	COL	Survived	–	COL/10	MTP/5
45.	TA	*A. baumannii *	ICU-1	2020/6/17	2020/6/28	2020/7/7	51	53	Pos	COL	Died	CKD	COL/18, TIG/12	DXM/22
		*K*. *pneumoniae*								COL				
	Bal	*K*. *pneumoniae*				2020/7/27				-				
46.	TA	*K*. *pneumoniae*	ICU-1	2020/6/13	2020/6/21	2020/6/27	23	28	Neg	COL	Died	–	COL/10	DXM/5, MTP/4
47.	Sputum	*A. baumannii *	ICU-1	2020/6/18	NI	2020/6/27	13	14	Neg	COL	Survived	-	COL/6	DXM/4
		*K*. *pneumoniae*								COL				
48.	TA	*A. baumannii *	ICU-1	2020/7/11	2020/7/11	2020/7/18	23	24	Neg	COL	Died	–	COL/18	–
		*K*. *pneumoniae*								COL				
49.	Sputum	*A. baumannii *	ICU-2	2020/7/9	NI	2020/7/20	7	11	Pos	AM, COL	Died	CKD, COPD	-	MTP/7
		*K*. *pneumoniae*								COL				
50.	TA	*A. baumannii *	ICU-2	2020/7/22	2020/8/5	2020/8/5	13	14	Pos	COL	Died	Heart disease	–	MTP/3
		*K*. *pneumoniae*								COL				
51.	TA	*K*. *pneumoniae*	ICU-1	2020/7/30	2020/8/3	2020/8/5	9	10	Pos	COL	Died	CKD	COL/6	DXM/3, MTP/3
52.	TA	*A. baumannii *	ICU-1	2020/7/28	2020/8/1	2020/8/7	10	12	Pos	COL	Died	Diabetes	–	MTP/4
		*K*. *pneumoniae*								COL				
53.	Sputum	*K*. *pneumoniae*	ICU-1	2020/7/17	NI	2020/8/8	25	24	Pos	AM, GM, PTZ, SAM, CAZ, LEV, MEM, COL,	Died	MI	MEM/7, LEV/7, COL/13	DXM/8
54.	TA	*A. baumannii *	ICU-1	2020/7/31	2020/7/31	2020/8/9	30	33	Neg	SAM, COL	Survived	–	COL/16	DXM/18
55.	TA	*A. baumannii *	ICU-1	2020/7/27	2020/8/4	2020/8/7	20	21	Pos	COL	Died	-	COL/7	MTP/4
	TA	*Enterobacter*				2020/8/13				PTZ, CAZ				
56.	TA	*A. baumannii *	ICU-2	2020/8/13	2020/8/18	2020/8/20	6	7	Neg	SAM, COL	Died	–	–	MTP/2
		*K*. *pneumoniae*								COL				
57.	Blood	*K*. *pneumoniae*	ICU-1	2020/7/29	NI	2020/8/21	22	23	Pos	COL	Died	-	COL/10	MTP/5
58.	TA	*K*. *pneumoniae*	ICU-1	2020/8/6	2020/8/18	2020/8/21	13	16	Pos	–	Died	Diabetes, Heart disease	COL/2	MTP/8
59.	TA	*K*. *pneumoniae*	ICU-2	2020/8/18	2020/8/21	2020/8/24	2	6	Pos	-	Died	-	-	-
60.	TA	*K*. *pneumoniae*	ICU-1	2020/8/19	2020/8/19	2020/8/24	10	22	Neg	CIP, LEV, COL	Survived	–	COL/14	DXM/8
61.	TA	*K*. *pneumoniae*	ICU-2	2020/8/14	2020/8/16	2020/8/25	16	17	Pos	COL	Died	-	COL/4	DXM/2, MTP/4
62.	TA	*K*. *pneumoniae*	ICU-1	2020/8/22	2020/8/27	2020/8/27	10	20	Pos	COL	Survived	Diabetes	COL/12	DXM/10
63.	TA	*K*. *pneumoniae*	ICU-1	2020/8/19	2020/8/19	2020/8/28	13	15	Pos	COL	Died	Diabetes	COL/3	MTP/2
64.	Sputum	*K*. *pneumoniae*	ICU-1	2020/8/10	NI	2020/8/31	19	22	Pos	COL	Died	Diabetes, Heart disease	COL/4	MTP/6
65.	TA	*A. baumannii *	ICU-1	2020/7/23	2020/8/27	2020/9/6	20	45	Pos	SAM, COL	Died	Diabetes, CKD	COL/14	DXM/15, MTP/4
		*K*. *pneumoniae*								COL				

Bal, Bronchoalveolar lavage; TA, Tracheal Aspirate; ICU, Intensive Care Unit; NI, Not Intubate; Pos, Positive; Neg, Negative; SAM, Ampicillin/sulbactam; COL, Colistin; AM, Amikacin; PTZ, Piperacillin-tazobactam; CAZ, Ceftazidime; MEM, Meropenem; GM, Gentamicin; VAN, Vancomycin; LIN, Linezolid; TEC, Teicoplanin; CIP, Ciprofloxacin; LEV, Levofloxacin; TIG, Tigecycline; CKD, Chronic Kidney disease; COPD, Chronic obstructive pulmonary disease; CND, Chronic neurological disease; MI, Medical immunosuppression; MTP, Methyl-prednisolone; DXM, Dexamethasone.

*In this column, only the antibiotics prescribed after the diagnosis of bacterial infections are listed.

In this study, of the 65 patients with secondary bacterial infection, 39 out of 65 (60%) had positive RT-PCR results and 53 out of 65 (81.5%) had a positive chest CT scan. Also, all 26 patients (40%) with negative RT-PCR had typical appearance [ground glass opacity (GGO)] on chest CT. In addition, consolidation and mixed (GGO pulse consolidation) in chest CT scan of patients were 12.3% (8/65) and 9.2% (6/65), respectively.

Out of a total of 365 samples (279 samples in ICU-1 and 86 samples in ICU-2) collected for microbiological culture, 70 (19.2%) and six (1.6%) samples were positive for bacterial and fungal growth, respectively. Out of the total positive samples tested for bacteria, 62/70 (88.6%) were respiratory specimens [endotracheal aspirate, sputum and bronchoalveolar lavage (BAL)], 6/70 (8.6%) were blood samples, 1/70 (1.4%) was a urine sample and 1/70 (1.4%) was a wound sample. Overall, 86 bacterial isolates were collected from 65 patients. Seventy-two strains (83.7%) were recovered from 54 patients in ICU-1, whereas the remaining isolates were recovered from 11 inpatients in ICU-2 ward. Also, out of six positive samples for fungal isolates, six *Candida* species were reported and all of them were collected from urine samples. According to culture results, the commonest co-pathogens identified were mainly Gram-negative bacteria (GNB), including *K. pneumoniae* (n = 47), *A. baumannii* (n = 35), *Enterobacter cloacae* (n = 1), *E. coli* (n = 1), and *Pseudomonas aeruginosa* (n = 1). Only one Gram-positive bacteria (*S. epidermidis*) was isolated. Mixed infection with more than one bacteria isolated from the same site was seen in 24.6% (16/65) of patients. The results are shown in **Table 2**. In this study, pneumonia was the most commonly reported bacterial infection (n = 62), followed by bacterial bloodstream infection (n = 6), bacterial wound infection (n = 1) and bacterial urinary tract infection (n = 1). It should be noted that some patients develop more than one infection during hospitalization in the ICU wards (see **Table 2**). Also, six fungal urinary tract infections caused by *Candida* species were observed.

In this study, thirty-eight patients were diagnosed with ventilator-associated pneumonia (VAP). Eighteen patients were early-onset VAP (EOVAP) and 20 patients were late-onset VAP (LOVAP) (see **Table 2**).

### Antimicrobial Susceptibility

Susceptibility profiles against eleven antimicrobials agents are listed in **Table 3**. Highest resistance was seen in *K. pneumoniae* isolates against nearly all antimicrobials agents. The rate of *K. pneumoniae* isolates exhibiting resistance to colistin was 10.6% (5/47), with MICs ranging from 4 to 16 mg/L. All *A. baumannii* isolates demonstrated resistance to meropenem, ceftazidime, cefotaxime, cefepime, piperacillin/tazobactam, fluoroquinolones and all of them were susceptible to colistin. The percentages of resistance to other antimicrobial agents among *A. baumannii* isolates were as follows: amikacin 94.3% (33/35); gentamicin 97.2% (34/35) and ampicillin/sulbactam 60% (21/35). *S. epidermidis* were resistant to all antimicrobial agents, with the exception of vancomycin, linezolid, and teicoplanin.

**Table 3:** Drug resistance rate of five GNB to antibiotics in patients with COVID-19 from January to April in Isfahan, Iran.

Antibiotics	*K*. *pneumoniae* (47)			*A. baumannii* (35)			*E. coli* (1)			*P. aeruginosa* (1)			*Enterobacter* spp. (1)		
	S%	I%	R%	S%	I%	R%	S%	I%	R%	S%	I%	R%	S%	I%	R%
**Ceftazidime**	2.1	0	97.9	0	0	100	100	0	0	0	0	100	100	0	0
**Ceftriaxone**	2.1	0	97.9	0	0	100	0	0	100	0	0	100	0	0	100
**Cefepime**	2.1	0	97.9	0	0	100	100	0	0	100	0	0	100	0	0
**Ampicillin/sulbactam**	2.1	0	97.9	34.3	5.7	60	0	0	100	0	0	100	0	0	100
**Piperacillin/tazobactam**	2.1	0	97.9	0	0	100	100	0	0	100	0	0	100	0	0
**Meropenem**	4.2	0	95.8	0	0	100	100	0	0	100	0	0	0	0	100
**Amikacin**	17	2.1	80.9	5.7	0	94.3	100	0	0	100	0	0	0	0	100
**Gentamicin**	6.4	0	93.6	2.8	0	97.2	100	0	0	100	0	0	0	0	100
**Ciprofloxacin**	2.1	0	97.9	0	0	100	0	0	100	0	0	100	0	0	100
**Levofloxacin**	4.2	0	95.8	0	0	100	0	0	100	0	0	100	0	0	100
**Colistin**	89.4	0	10.6	100	0	0	100	0	0	100	0	0	0	0	100

### Patterns of Antibiotic Use and Antiviral Therapy

Thirty-six of the 65 (55.4%) of patients received antimicrobial therapy within 24 h of admission: in twenty-five patients antimicrobial therapy was initiated in the emergency department, and in 11 patients therapy was initiated on ICU (in cases of direct admittance to ICU). In terms of antibiotic prescribing patterns, 100% (n = 65) of all patients received at least two antibiotics. The most frequently used antibiotic regimen was meropenem (56/65 86.2%) with a median duration of 12 (2–32) days, followed by levofloxacin (48/65 70.8%), colistin (44/65 67.7%), linezolid (40/65 61.5%), vancomycin (31/65 47.7%), with a median duration of 9 (2–24), 8 (1–34), 10 (2–14) and 7 (2–14) days, respectively. Detailed information on antibiotic therapy including duration of therapy is presented in **Table 4**. Also, among 65 patients with superinfection, 38.4, 4.6, and 3% patients received hydroxychloroquine, oseltamivir, and kaletra, respectively (**Table 1**).

**Table 4:** Most commonly used antibiotics for treatment of patients with secondary bacterial infection.

Use of empirical antibiotics, n (%)	Value (n = 65)
Use of more than one class of empirical antibiotics, n (%)	65 (100%)
Cephalosporin (Ceftazidime, Ceftriaxone), n (%)	13 (20%)
Duration of therapy (days)	7 (2–15)
Azithromycin, n (%)	7 (10.7%)
Duration of therapy (days)	4 (3–6)
Aminoglycosides (Amikacin, Tobramycin), n (%)	4 (6%)
Duration of therapy (days)	7 (5–10)
Levofloxacin, n (%)	48 (73.8%)
Duration of therapy (days)	9 (2–24)
Piperacillin/tazobactam, n (%)	14 (21.5%)
Duration of therapy (days)	8 (3–12)
Ampicillin/sulbactam, n (%)	5 (7.7%)
Duration of therapy (days)	8 (4–14)
Meropenem, n (%)	56 (86.2%)
Duration of therapy (days)	12 (2–32)
Colistin, n (%)	44 (67.7%)
Duration of therapy (days)	8 (1–34)
Vancomycin, n (%)	31 (47.7%)
Duration of therapy (days)	7 (2–14)
Linezolid, n (%)	40 (61.5%)
Duration of therapy (days)	10 (2–14)

## Discussion

This study belongs to a group of early microbiological studies that report clinical characteristics and outcomes in COVID-19 patients admitted to the ICUs with secondary bacterial infections in Iran. The main results are as follows: (1) 11.9% of the COVID-19 patients admitted to our ICUs have a superinfection upon ICU admission; (2) the leading involved bacteria were extremely drug-resistant (XDR) GNB specially *K. pneumoniae* and *A. baumannii*; (3) inappropriate use or overuse of antibiotics and change in antimicrobial use during the pandemic. At present, there are limited data regarding bacterial co-infection and secondary infection in COVID-19 in Iran. However, in a study carried out by Sharifipour et al. in Qom, Iran, it was reported that bacterial co-infection with *A. baumannii* and *Staphylococcus aureus* identified in nineteen COVID-19 patients admitted to the ICU (Sharifipour et al., 2020). It is important to mention that all data collected and analyzed in this study is related to pre tocilizumab era.

The prevalence of bacterial infections in COVID-19 patients admitted to the ICU has been reported in several studies. (Contou et al., 2020; Sharifipour et al., 2020; Baskaran et al., 2021). A recent systematic review and meta-analysis study evaluating co-infections among patients infected with COVID-19 performed by Langford et al. reported that the rate of secondary bacterial infection is 14.3% (Langford et al., 2020). Recent studies reported 41 and 28% rates of co-infection among patients admitted to a North American and French ICUs (Contou et al., 2020; Lehmann et al., 2020). Respiratory sites were the most common sites of bacterial infection in COVID-19 patients. Gram-negative pathogens were predominant in respiratory infections. Herein, we report on a 11.9% rate of bacterial superinfection mostly due to carbapenem resistant *K. pneumoniae* (CRKP) and *A. baumannii* (CRAB). This observation was similar to the findings reported by previous studies that investigated bacterial co-infections in patients with COVID-19, particularly ICU cohorts (Contou et al., 2020; Sharifipour et al., 2020; Baskaran et al., 2021). In this COVID-19 pandemic, Yang et al. have reported that bacterial co-infections were found in around 11.5% COVID-19 patients, which is in accordance with our results (11.9%) (Yang et al., 2020). According to recent studies in Iran, CRKP and CRAB had the highest rates of incidence in ICUs (Solgi et al., 2017; Sharifipour et al., 2020; Solgi et al., 2020; Bolourchi et al., 2021; Nazari et al., 2021).

During the study period, 38 patients were diagnosed with VAP. Eighteen patients were EOVAP and 20 patients were LOVAP (see **Table 2**). Pathogens from this study did not differ between EOVAP and LOVAP. The results of our study were similar to other tertiary centers in India and Thailand (Arayasukawat et al., 2012; Jakribettu et al., 2016). In addition, we observed 6 cases of fungal co-infections caused by *Candida* species. In a systematic review in 2020, Lansbury and colleagues have reported that the rate of fungal infections was low among COVID-19 patients, which is in line with our results (Lansbury et al., 2020).

The median length of ICU stay among patients in our study was high, 17 days (4 to 61), findings similar to the study of Sharifipour et al. (2020), which reported a length of stay of 15 days (2–39) of all patients with COVID-19 admitted to their ICU. In this study, there was a significantly higher rate of inpatient mortality (83% 54/65) among patients with secondary bacterial infection compared to those without superinfection (211–54/488 = 32.1%). Among patients admitted to ICU wards, we found an association between patients identified to have superinfection and inpatient mortality on univariable analysis (P < .0001). In a study conducted by Neto and colleagues on COVID-19 patients with secondary bacterial infections in the United States, mortality rate was 50% (OR, 5.838; P <.0001) (Neto et al., 2020). Similarly, the study by Vijay and colleagues in COVID-19 cases indicated that the mortality among patients who acquired secondary infections was 56.7% (Vijay et al., 2021).

Despite an overall low rate of bacterial infections in our study, all of 65 patients who acquired bacterial infection received at least two empiric antibiotic therapy upon ICU admission before first positive MDR-GNB culture, with the majority constituting broad-spectrum agents such as levofloxacin, meropenem and linezolid, which can cause the lack of growth of not-MDR pathogens in culture samples. Similarly, the study by Patel and colleagues in COVID-19 cases indicated that of the 71 patients who had MDR GNB infection, 69 (97%) had received antibiotics before first positive resistant GNB culture (Patel et al., 2021). Moreover, these findings are similar to a study in New York reported empirical antibiotics use among 79% of COVID-19 inpatients (Nori et al., 2020) and several studies performed during the beginning of the COVID-19 pandemic from China have reported that over 90% of patients received empirical antibiotics (Wang et al., 2020; Wu et al., 2020). In the present study, GNB were the most commonly recovered organisms from specimen cultures in patients with COVID-19. The predominance of GNB in this study likely reflects nosocomial infection following prolonged ICU stay and empirical antibiotic use.

The frequent prescription of broad-spectrum empirical antimicrobials in COVID-19 patients could increase antibiotic resistance in the near future. Therefore, strict programs of antimicrobial stewardship are required to improve antibacterial use.

The predominance of CRKP and CRAB isolates in current study, could be due to the invasive device-associated infections during ICU stay due to mechanical ventilation and empirical antibiotic use. Also indicated was an environmental source, pointing to poor hand hygiene and poor infection control practices.

During COVID-19 pandemic, due to fear of COVID-19 approximately all healthcare workers use gloves as part of personal protective equipment and do not feel the need to perform hand hygiene before and after patient care, and there is a lack of concern for patient to patient transmission of XDR pathogens in hospitalized patients (Vijay et al., 2021). As most of the bacterial infections in the present study were XDR pathogens nosocomial in origin, it highlighted poor infection control measures (e.g., hand washing, glove changing, and meticulous cleaning of equipment), that could have led to cross-transmission of XDR pathogens during patient care.

Our study has several limitations. First, it is a single-center retrospective study from an academic hospital in the epicenter of the COVID-19 pandemic, and the data collected were limited. Therefore, our results may not be generalizable to other centers with a different bacterial ecology. Second, during the study period, molecular diagnostic tests (e.g., PCR) were not available for detection of atypical pathogens, such as *Mycoplasma pneumoniae*. Therefore, we might have missed atypical secondary bacterial infection identified and other respiratory pathogen infections usually diagnosed by PCR. Third, due to the lack of a mycology laboratory in our hospital, it was not possible to accurately identify fungal infections. So, the true incidence of fungal infections remains unclear. Finally, a differential diagnosis of GGO pattern was not performed.

In conclusion, the superinfection among patients admitted to the ICU wards is a serious problem in the COVID-19 pandemic, which can lead to increasing the disease severity and mortality. Herein, we report on a 11.9% rate of secondary bacterial infection at ICU admission of adult patients with severe COVID-19, mostly with CRKP and CRAB that associated with high mortality rates (83%). These data are also in line with the previously documented high mortality associated with these pathogens (Balkhair et al., 2019). With increasing cases of COVID-19 in Iran, there is a need to reinforce the principles of antimicrobial stewardship and infection control.

## Data Availability Statement

The original contributions presented in the study are included in the article/supplementary material. Further inquiries can be directed to the corresponding author.

## Ethics Statement

The studies involving human participants were reviewed and approved by No. IR.MUI.MED.REC.1400.453. Written informed consent for participation was not required for this study in accordance with the national legislation and the institutional requirements.

## Author Contributions

Project design: HS, SP and EK. Investigation: HS, MS and MA. Data analysis: HS. Writing—Original draft: HS, HT, HM, FS, and BA. Writing—Review & editing: HS, SP, EK, HT, HM, RS, BA, and FK. All authors listed have made a substantial, direct, and intellectual contribution to the work and approved it for publication.

## Funding

This work was funded by research grants from the Isfahan University of Medical Sciences (project no. 50485).

## Conflict of Interest

The authors declare that the research was conducted in the absence of any commercial or financial relationships that could be construed as a potential conflict of interest.

## Publisher’s Note

All claims expressed in this article are solely those of the authors and do not necessarily represent those of their affiliated organizations, or those of the publisher, the editors and the reviewers. Any product that may be evaluated in this article, or claim that may be made by its manufacturer, is not guaranteed or endorsed by the publisher.

## References

[B1] Arayasukawat P. So−Ngern A. Reechaipichitkul W. Chumpangern W. Arunsurat I. Ratanawatkul P.. (2012). Microorganisms and Clinical Outcomes of Early- and Late-Onset Ventilator-Associated Pneumonia at Srinagarind Hospital, a Tertiary Center in Northeastern Thailand. BMC Pulm Med. 21, 1–8. doi: 10.1186/s12890-021-01415-8 PMC7847239 33516213

[B2] Balkhair A. Al-Muharrmi Z. Al’adawi B. Al Busaidi I. Taher H. B. AL-Siyabi T.. (2019). Prevalence and 30-Day All-Cause Mortality of Carbapenem-and Colistin-Resistant Bacteraemia Caused by Acinetobacter Baumannii, Pseudomonas Aeruginosa, and Klebsiella Pneumoniae: Description of a Decade-Long Trend. Int. J. Infect. Dis. 85, 10–15. doi: 10.1016/j.ijid.2019.05.004 31100418

[B3] Baskaran V. Lawrence H. Elansbury L. Webb K. Safavi S. Zainuddin N.. (2021). Co-Infection in Critically Ill Patients With COVID-19: An Observational Cohort Study From England. J. Med. Microb. 70, 1–9. doi: 10.1099/jmm.0.001350 PMC8289210 33861190

[B4] Bolourchi N. Shahcheraghi F. Giske C. G. Nematzadeh S. Noori Goodarzi N. Solgi H.. (2021). Comparative Genome Analysis of Colistin-Resistant OXA-48-Producing *Klebsiella Pneumoniae* Clinical Strains Isolated From Two Iranian Hospitals. Ann. Clin. Microbiol. Antimicrob. 74, 1–11. doi: 10.1186/s12941-021-00479-y PMC8542297 34688302

[B5] Clinical and Laboratory Standards Institute [CLSI] (2017). Performance Standards for Antimicrobial Susceptibility Testing. 27th Edn (Wayne, PA: Clinical and Laboratory Standards Institute).

[B6] Contou D. Claudinon A. Pajot O. Micaëlo M. Flandre P. L. Dobert M.. (2020). Bacterial and Viral Co-Infections in Patients With Severe SARS-CoV-2 Pneumonia Admitted to a French ICU. Ann. Intensive Care 10, 1–9. doi: 10.1186/s13613-020-00736-x 32894364 PMC7475952

[B7] Corman V. M. Landt O. Kaiser M. Molenkamp R. Meijer A. Chu D. K. W.. (2020). Detection of 2019 Novel Coronaviru -Ncov) by Real-Time RT-PCR. Euro Surveill 25, 2000045. doi: 10.2807/1560-7917.ES.2020.25.3.2000045 PMC6988269 31992387

[B8] EUCAST. (2017). Breakpoint Tables for Interpretation of MICs and Zone Diameters. Version 7.0. Available at: http://www.eucast.org/clinical_breakpoints/ (Accessed February 24, 2017).

[B9] Jakribettu R. Boloor R. Suresh S. (2016). Comparison of Microbiological Profile of Pathogens Isolated From Early−Onset and Late−Onset Ventilator−Associated Pneumonia in a Tertiary Care Center. Trop. J. Med. Res. 19, 14–19. doi: 10.4103/1119-0388.172073

[B10] Langford B. So M. Raybardhan S. Leung V. Westwood D. MacFadden D. R.. (2020). Bacterial Co-Infection and Secondary Infection in Patients With COVID-19: A Living Rapid Review and Meta-Analysis. Clin. Microbiol. Infect. 26, 1622–1629. doi: 10.1016/j.cmi.2020.07.016 32711058 PMC7832079

[B11] Lansbury L. Lim B. Baskaran V. Lim W. S. (2020). Co-Infections in People With COVID-19: A Systematic Review and Meta-Analysis. J. Infect 81, 266–275. doi: 10.1016/j.jinf.2020.05.046 32473235 PMC7255350

[B12] Lehmann C. J. Pho M. T. Pitrak D. Ridgway J. Pettit N. N.. (2020). Community Acquired Co-Infection in COVID-19: A Retrospective Observational Experience. Clin. Infect. Dis. 72, 1450–1452. doi: 10.1093/cid/ciaa902 PMC7337635 32604413

[B13] Nazari M. Azizi O. Solgi H. Fereshteh S. Shokouhi S. Badmasti F. (2021). Emergence of Carbapenem Resistant Acinetobacter Baumannii Clonal Complexes CC2 and CC10 Among Fecal Carriages in an Educational Hospital. Int. J. Environ. Health Res. 15, 1–12. doi: 10.1080/09603123.2021.1892036 33855919

[B14] Neto A. G. M. Bryan L. O. K. Wattoo A. Salacup G. Pelayo J. Dejoy R.. (2020). Bacterial Infections and Patterns of Antibiotic Use in Patients With COVID - 19. J. Med. Virol. 18, 1–7. doi: 10.1002/jmv.26441 PMC7461450 32808695

[B15] Nori P. Cowman K. Chen V. Bartash R. Szymczak W. Madaline T.. (2020). Bacterial and Fungal Coinfections in COVID-19 Patients Hospitalized During the New York City Pandemic Surge. Infect. Control Hosp. Epidemiol. 42, 84–88. doi: 10.1017/ice.2020.368 32703320 PMC7417979

[B16] Patel A. Emerick M. Cabunoc M. K. Williams M. H. Preas M. A. Schrank G.. (2021). Rapid Spread and Control of Multidrug-Resistant Gram-Negative Bacteria in COVID-19 Patient Care Units. Emerg. Infect. Dis. 27, 1234–1236. doi: 10.3201/eid2704.204036 PMC8007317 33565961

[B17] Russell C. D. Fairfield C. J. Drake T. M. Turtle L. Seaton A. Wootton D. G.. (2021). Co-Infections, Secondary Infections, and Antimicrobial Use in Patients Hospitalised With COVID-19 During the First Pandemic Wave From the ISARIC WHO CCP-UK Study: A Multicentre, Prospective Cohort Study. Lancet Microbe. 2, e354–65. doi: 10.1016/S2666-5247(21)00090-2 PMC8172149 34100002

[B18] Sharifipour E. Shams S. Esmkhani M. Khodadadi J. Fotouhi-Ardakani R. Koohpaei A.. (2020). Evaluation of Bacterial Co-Infections of the Respiratory Tract in COVID-19 Patients Admitted to ICU. BMC Infect. Dis. 20, 1–7. doi: 10.1186/s12879-020-05374-z PMC7461753 32873235

[B19] Solgi H. Ghafarzadeh H. Shahcheraghi F. (2017). Evaluation of Phenotypic and Genotypic Carbapenemase Genes in Gram-Negative Bacteria Resistant to Carbapenem and Determining Their Antibiotic Resistance. J. Isfahan Med. Sch. 34, 1290–1296.

[B20] Solgi H. Shahcheraghi F. Bolourchi N. Ahmadi A.. (2020). Molecular Characterization of Carbapenem-Resistant Serotype K1 Hypervirulent Klebsiella Pneumoniae ST11 Harbouring bla_NDM_-_1_ and *bla* _OXA_ *-* _48_ Carbapenemases in Iran. Microb. Pathog. 149, 104507. doi: 10.1016/j.micpath.2020.104507 32950637

[B21] Turton J. F. Woodford N. Glover J. Yarde S. Kaufmann M. E. Pitt T.. (2006). Identification of Acinetobacter Baumannii by Detection of the Bla OXA-51 -Like Carbapenemase Gene Intrinsic to This Species. J. Clin. Microbiol. 44, 2974–2976. doi: 10.1128/JCM.01021-06 16891520 PMC1594603

[B22] Vijay S. Bansal N. Kumar Rao B. Veeraraghavan B. Rodrigues C. Wattal C.. (2021). Secondary Infections in Hospitalized COVID-19 Patients: Indian Experience. Infect. Drug Resist. 14, 1893–1903. doi: 10.2147/IDR.S299774 34079300 PMC8164345

[B23] Wang Z. Yang B. Li Q. Wen L. Zhang R.. (2020). Clinical Features of 69 Cases With Coronavirus Disease 2019 in Wuhan, China. Clin. Infect. Dis. 71, 769–777. doi: 10.1093/cid/ciaa272 32176772 PMC7184452

[B24] WHO. Covid-19 Dashboard 2020. Available at: https://covid19.who.int/?gclid=CjwKCAjw8df2BRA3EiwAvfZWaJWnmCWZBUjJdJZGVdH4hGENu8orjqQTHDsIst5u_gYXoQcl8sS_ZxoCIxEQAvD_BwE.

[B25] World Health Organization. (2020). Clinical Management of Severe Acute Respiratory Infection (SARI) When COVID-19 Disease is Suspected: Interim Guidance. Available at: https://www.who.int/publications-detail/clinical-management-of-severe-acuterespiratory-infection-when-novel-coronavirus-(ncov)-infection-is-suspected (Accessed Mar 29, 2020).

[B26] Wu J. Liu J. Zhao X. Liu C. Wang W. Wang D.. (2020). Clinical Characteristics of Imported Cases of Coronavirus Disease 2019 (COVID-19) in Jiangsu Province: A Multicenter Descriptive Study. Clin. Infect. Dis. 71, 706–712. doi: 10.1093/cid/ciaa199 32109279 PMC7108195

[B27] Yang X. Yu Y. Xu J. Shu H. Xia J. Liu H.. (2020). Clinical Course and Outcomes of Critically Ill Patients With SARS-CoV-2 Pneumonia in Wuhan, China: A Single-Centered, Retrospective, Observational Study. Lancet Respir. Med. 8, 475–481. doi: 10.1016/S2213-2600(20)30079-5 32105632 PMC7102538

